# Prediction model for milk transfer of drugs by primarily evaluating the area under the curve using QSAR/QSPR

**DOI:** 10.1007/s11095-023-03477-1

**Published:** 2023-01-31

**Authors:** Tae Maeshima, Shin Yoshida, Machiko Watanabe, Fumio Itagaki

**Affiliations:** Department of Clinical & Pharmaceutical Sciences, Faculty of Pharma Science, Teikyo University, Itabashi-Ku, Tokyo, 173-8605 Japan

**Keywords:** active transport, ADMET predictor®, *in silico*, M/P_AUC_, QSAR

## Abstract

**Purpose:**

Information on milk transferability of drugs is important for patients who wish to breastfeed. The purpose of this study is to develop a prediction model for milk-to-plasma drug concentration ratio based on area under the curve (M/P_AUC_). The quantitative structure–activity/property relationship (QSAR/QSPR) approach was used to predict compounds involved in active transport during milk transfer.

**Methods:**

We collected M/P ratio data from literature, which were curated and divided into M/P_AUC_ ≥ 1 and M/P_AUC_ < 1. Using the ADMET Predictor® and ADMET Modeler™, we constructed two types of binary classification models: an artificial neural network (ANN) and a support vector machine (SVM).

**Results:**

M/P ratios of 403 compounds were collected, M/P_AUC_ data were obtained for 173 compounds, while 230 compounds only had M/P_non-AUC_ values reported. The models were constructed using 129 of the 173 compounds, excluding colostrum data. The sensitivity of the ANN model was 0.969 for the training set and 0.833 for the test set, while the sensitivity of the SVM model was 0.971 for the training set and 0.667 for the test set. The contribution of the charge-based descriptor was high in both models.

**Conclusions:**

We built a M/P_AUC_ prediction model using QSAR/QSPR. These predictive models can play an auxiliary role in evaluating the milk transferability of drugs.

**Supplementary Information:**

The online version contains supplementary material available at 10.1007/s11095-023-03477-1.

## Introduction

Breastfeeding is known to have various advantages, such as improving the immune [1] and cognitive functions of the infant [2], reducing the prevalence of certain diseases in the future [3], reducing the risk of various diseases in the mother [4, 5], and building a good mother-infant relationship [6]. Due to these reasons, the World Health Organization recommends exclusive breastfeeding [7]. According to a 2015 survey in Japan, over 90% of pregnant women chose to breastfeed over other alternatives [8]. However, with advancement in mothers’ age at the time of childbirth and improved treatment for pregnancy complications, the number of women receiving drug treatment during their pregnancy and delivery is increasing [9]. Even if a pregnant woman on medication wishes to breastfeed, there is insufficient information available regarding the milk transferability of drugs. Moreover, it is ethically challenging to conduct human clinical trials to assess the milk transferability of drugs. Several factors affecting this transferability are known, including molecular weight, pH, lipophilicity, and plasma protein-binding [10]. Milk transfer of drugs involves passive diffusion. However, some drugs have also been shown to be actively carried by transporters, such as breast cancer resistance protein (BCRP) [11].

The milk-to-plasma drug concentration (M/P) ratio is an indicator of the transferability of drugs to milk. The safety of drug therapy for nursing mothers cannot be evaluated based on the M/P ratio alone; maternal and infant clearance and other factors must be considered. The M/P ratio is also used to calculate relative infant dose (RID) and exposure index (EI), which are pharmacokinetic measures [12]. These indicators can help determine the precise amount of drug ingested by the child. This is important to evaluate whether breastfeeding can be combined with drug therapy, making these indicators helpful in drug therapy for lactating women [13]. Recently, the M/P ratio has been used as a parameter in the *in silico* Physiologically based pharmacokinetic model, contributing to the development of drug therapy for lactating women [14].

Prediction of M/P ratios has been attempted since the 1980s, when a phase distribution model was reported to predict M/P ratios from the physicochemical properties of compounds [15, 16]. These were noted for their independence from clinical data and the fact that they did not take into account the effects of active transport [14]. Subsequently, an M/P prediction model using quantitative structure-activity relationships (QSAR/QSPR) was reported. The QSAR/QSPR approach aims to find correlations between structural features or physicochemical constants of a drug and its biological activity, and can be applied to predict physical and chemical properties by means of descriptors that explain changes in the physical or chemical properties of that drug group. A number of linear regression models have been reported for QSAR models predicting M/P ratios [17–23], but because M/P ratio data are collected from individual reports, uncertainties in subjects, measurement methods, and variations in the number of cases may affect the models. A classification model was also constructed based on the idea that prediction by linear regression is not realistic [24, 25]. Even with the establishment of highly accurate models, predicting milk transferability of actively transported drugs remains a challenge [14].

It is important to have organized and curated data for QSAR/QSPR model building. Datasets from previously reported models included inconsistent M/P ratios for animals and inconsistent sampling times for milk and plasma. Colostrum and mature milk also differ in pH, fat content, and secretion, but were not necessarily separated in the dataset. In addition, it is more appropriate to evaluate the transfer of a drug to milk using the area under the curve (AUC) (M/P_AUC_) rather than its concentration at a specific time point. This is because drug concentrations in maternal plasma and breast milk are not always in equilibrium. Furthermore, some drugs have been reported to take a longer time to reach equilibrium [26, 27]. However, frequent sampling for AUC calculation in clinical practice is not easy, and M/P_AUC_ is not often reported. M/P_AUC_ should be used to evaluate milk transfer.

The purpose of this study was to curate M/P ratio data and build a binomial classification model based on M/P_AUC_ for screening drugs involved in active transport in human mature milk.

## Materials and Methods

### Datasets

Human M/P ratio data were obtained from original papers with reference to various sources such as books [28, 29] and the LactMed database [30]. Only data from mature milk were extracted; data from colostrum up to 7 days postpartum were excluded. Only data evaluated by AUC were used for M/P ratios for model building, and M/P ratios calculated by other methods such as single point evaluation were excluded as M/P_non-AUC_. Reports of misplaced timing between breast milk and maternal blood samples were also excluded. When using data for which the M/P ratio was measured at multiple sampling times but the AUC was not calculated, the trapezoidal method was used to calculate the AUC. When only blood and milk drug concentration graphs were reported, these graphs were reproduced and AUCs were calculated. Protein preparations and metal-containing compounds whose physical properties could not be predicted by ADMET predictor^®^ were excluded. A comprehensive list of selected original articles for each compound can be found in Online Resource.

### Descriptors

The simplified molecular input line entry system (SMILES) format of the compounds was obtained from the public database PubChem [31]. These were incorporated into the ADMET predictor^®^ and 254 descriptors were generated. Descriptors belonging to the following categories were generated: simple constitutional descriptors, topological indices, atom-type electrophysiological state indices, charge-based descriptors, hydrogen bonding descriptors, molecular ionization descriptors, functional groups, Moriguchi descriptors, pattern-recognition flags, and Meylan flags. In contrast, the following categories were excluded: textual description, indicators, and 3D descriptors. The ADMET Modeler^TM^ allows the user to add any descriptor from among the 48 descriptors for the calculated or simulated physical properties of the compounds in ADMET Predictor^®^, in addition to the 254 molecular descriptors during model construction. The choice of descriptors used to build the model was made in two steps. First, descriptors were reduced in an unsupervised process based on their characteristics and relevance to other descriptors. Descriptors were eliminated based on three conditions: a coefficient of variation lower than the Minimum Coefficient variation setting; a non-zero value lower than the Minimum representation; and a Maximum absolute with the least amount of information when a pair of descriptors shows a higher absolute correlation coefficient than the value set in "Max absolute correlation".

This was followed by a supervised prioritization based on sensitivity. This was done using the functions 'Input Gradient', 'Truncated Linear analysis', 'Iterative truncated linear analysis', and 'Genetic algorithm'. Descriptor selection settings are described in the following sections for each model.

### Artificial neural network (ANN) model settings

Multilayer perceptron was used as the architecture model in the ANN. The Kohonen self-organizing map method was used for the test set selection [32]. That is, using the Kohonen map method, the compounds in the data set are divided into three sets: training set, test set, and validation set. This is a method of selecting test sets from cells in a toroidal two-dimensional Kohonen map that clusters compounds by chemical similarity in a descriptor space. The Kohonen size was set automatically and the Kohonen map was not reused. The minimum test set size was 10%. The settings for descriptor number reduction were minimal coefficient of variation: 1, minimum representation: 4, and maximum absolute correlation: 0.98. Sensitivity analysis was performed using truncated linear analysis. One Monte Carlo attempt was applied, and the maximum weight of data was 75%. In each ensemble, 33 individual networks were used and the network multiplier was 5. This number multiplied by the ensemble size represents the total number of networks trained per architecture. The Autofill function was used to set the minimum, maximum, and step values of hidden neurons and network inputs.

### Support vector machine (SVM) model setting

The support vector machine was used as the architecture model. The Kohonen map method was used for the test set selection. The Kohonen size was set automatically and the Kohonen map was not reused. The minimum test set size was 10%. The settings for descriptor number reduction were minimal coefficient of variation: 1, minimum representation: 4, and maximum absolute correlation: 0.98. In the SVM model, the descriptor "S+log D,” which is the value of log D predicted by the ADMET predictor^®^, was added manually. When calculating log D, the pH of the maternal blood was set at 7.4. Sensitivity analysis was performed using a genetic algorithm (GA) [33]. The target on which the genetic algorithm is executed can be selected from the grid, row, or cell. Because the cell level is recommended for small datasets, GA was run by the cell. The GA’s max steps was 30,000, and its max training was 1,000. The number of individual SVM models used in each ensemble was 33. The total number of SVM models used to train the ensembles was set to 40. These are both default settings in the software.

### Evaluation of the model using a confusion matrix

**Fig. 1:**
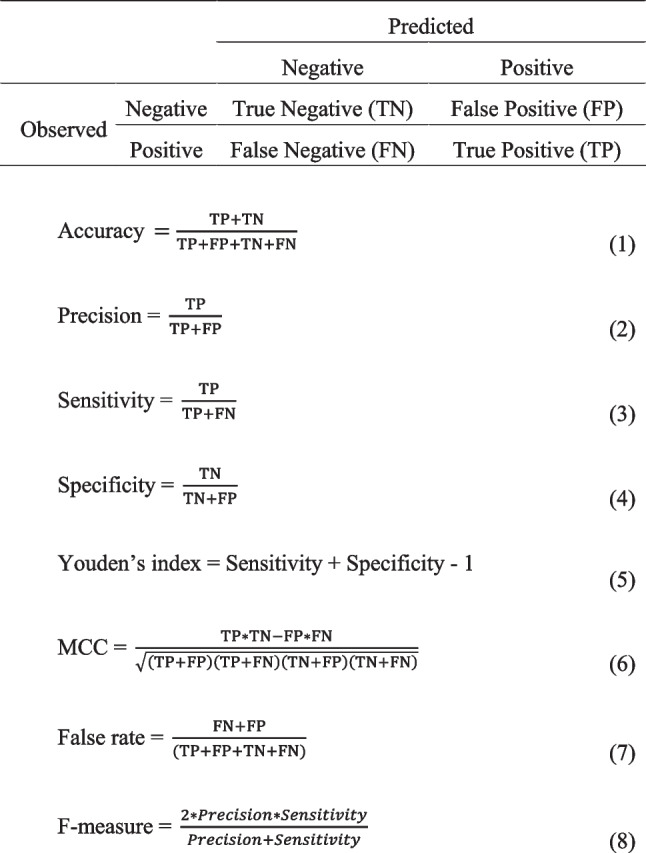
Confusion matrix and formulas for each indicator.

To evaluate the model, each index was calculated using a confusion matrix (Fig. 1).

### Software

DigitizeIt version 2.3.3 (I. Bormann, Braunschweig, Germany) was used to plot blood and milk concentration curves. ADMET Predictor^®^ version 10.3 (Simulations Plus Inc., Lancaster, CA, USA) was used to generate the molecular descriptors. Furthermore, ADMET Modeler^TM^ version 10.3 (Simulations Plus Inc., Lancaster, CA, USA) was used to build the model.

## Results

The M/P ratios of 403 compounds were obtained from literature. Of these, M/P_AUC_ was reported for 173 compounds, and data for 21 compounds were calculated by replotting the graph. Data for the remaining 230 compounds were obtained using the M/P_non-AUC_. Of the 173 compounds with M/P_AUC_ data, 129 compounds, excluding colostrum, were used in the dataset.

### ANN model performance

**Fig. 2:**
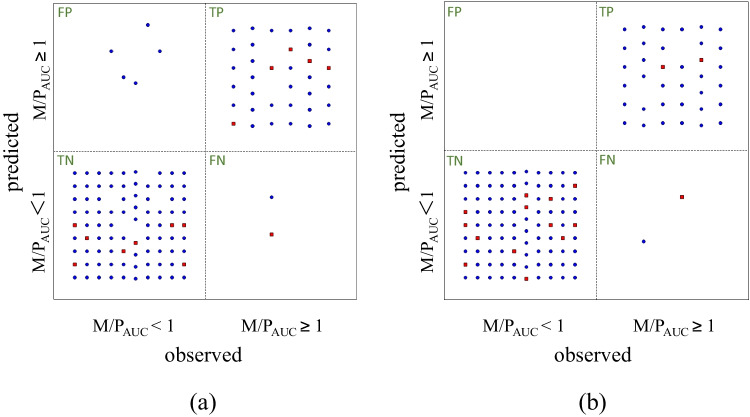
Statistical performance results for ANN model (**a**) and SVM model (**b**) using the ADMET Modeler™. Blue circles: training set data; red squares: test set data. FP, False positive; TP, True positive; TN, True negative; FN, False negative.

Of the 254 descriptors, 37 under-represented descriptors and 40 highly correlated descriptors were excluded. The remaining 177 descriptors were used to build the model as candidate inputs. Of the 129 compounds, 76 were assigned to the training set, 39 to the verification set, and the remaining 14 to the test set. The best model was made using 33 ANNs, 3 neuron, 24 inputs, and 79 weights. Table I lists the effect indicators for model evaluation. The sensitivity was 0.969 for the training set and 0.833 for the test set. The specificity was 0.940 for the training set and 1.000 for the test set. Furthermore, the Matthews correlation coefficient (MCC) was 0. 878 for the training set and 0.861 for the test set. The statistical performance results are shown in Fig. 2a. As shown in Table II, the most contributing descriptor in the ANN model was EEM_XFC. This descriptor is classified as a Charge-based Descriptor. Next was T-RDmtr. The third was Pi_AQc, which is also a Charge-based Descriptor. As an example, acetaminophen was predicted by the ANN model to have M/P_AUC_ ≥ 1 with 95% confidence; the sensitivity of EEM_XFc was -0.53, that of T_RDmtr was -0.77, and that of Pi_AQc was 0.71. On the other hand, for morphine, which is predicted to have M/P_AUC_ ≥ 1 with 65% confidence, EEM_XFc was -0.30, T_RDmtr was -0.64, and Pi_AQc was 0.70, with acetaminophen having the greater absolute value. The maximum uncertainty was 57.5%.

**Table I:** Model Output Statistics of ANN Model and SVM Models

Models	Data set	Accuracy	Precision	Sensitivity	Specificity	Youden	MCC	False rate	F-measure
ANN	Training	0.948	0.857	0.969	0.940	0.909	0.878	0.052	0.910
	Test	0.929	1.000	0.833	1.000	0.833	0.861	0.071	0.909
SVM	Training	0.991	1.000	0.971	1.000	0.971	0.979	0.009	0.985
	Test	0.938	1.000	0.667	1.000	0.667	0.787	0.063	0.800

ANN, artificial neural network; SVM, support vector machine; Youden, Youden index; MCC, Matthews correlation coefficient

**Table II:** Descriptors with High Contributions to Binary Classification Model

ANN model					SVM model				
Index	Name	Description	Sensitivity	Relative Sensitivity	Index	Name	Description	Sensitivity	Relative Sensitivity
1	EEM_XFc	Maximum sigma Fukui index on C	0.572	1.000	1	SaaaC	Atom-type E-state index for aCaa groups	2.268	1.000
2	T_RDmtr	Relative topological diameter: maximal topological distance divided by the number of atoms	0.468	0.817	2	EEM_NFpl	Minimum sigma Fukui index on polar atoms	2.261	0.997
3	Pi_AQc	Sum of absolute values of Hückel pi atomic charges, but only on C atoms	0.450	0.786	3	EEM_XFon	Maximum sigma Fukui index on N and O	2.250	0.992
4	Pi_FPl4	Fourth component of the autocorrelation vector of pi Fukui( +) indices	0.429	0.750	4	T_RDmtr	Relative topological diameter: maximal topological distance divided by the number of atoms	2.250	0.992
5	MinQ	Minimal PEOE Partial Atomic Charge	0.404	0.706	5	S + logD	octanol–water distribution coefficient (log D) calculated from S + pKa and S + logP	2.243	0.989
6	F_DbleB	Double bonds as fraction of total bonds	0.397	0.694	6	NPA_Q3	Third component of the autocorrelation vector of estimated NPA partial atomic charges	2.239	0.987
7	T_Radb	Topological equivalent of Radb__3D	0.381	0.666	7	EEM_XFc	Maximum sigma Fukui index on C	2.236	0.986
8	ArHdrxl_-OH	Number of aromatic hydroxyl groups	0.323	0.565	8	SecAmine_ > NH	Number of primary and aliphatic N secondary amines	2.225	0.981
9	EEM_XFpl	Maximum sigma Fukui index on polar atoms	0.315	0.551	9	EEM_NFnp	Minimum sigma Fukui index on nonpolar atoms	2.218	0.978
10	T_Rada	Topological equivalent of Rada__3D	0.311	0.543	10	NPA_MinQ	Minimal Estimated NPA Partial Atomic Charge	2.186	0.964

### SVM model performance

Of the 255 descriptors, 37 under-represented and 40 highly correlated descriptors were excluded. The remaining 178 descriptors were used to build the model as candidate inputs. Of the 129 compounds, 72 were assigned to the training set, 41 to the verification set, and the remaining 16 to the test set. The best SVM ensemble model uses 72 inputs. Table I lists the effect indicators for model evaluation. The sensitivity for the training set was 0.971 and 0.667 for the test set. The specificity was 1.000 for the training set and 1.000 for the test set. Moreover, the MCC was 0.979 for the training set and 0.787 for the test set. The statistical performance results are shown in Fig. 2b. Table II lists the top 10 descriptors with the highest contribution. The descriptor with the highest contribution in the SVM model was SaaaC, which is an Atom-type Electropological State Index. This is followed by EEM_NFpl and EEM_XFon, which are Charge-based Descriptors. EEM_XFc and T_RDmtr, the first and second highest contributors in the ANN model, were in the fourth and seventh positions in the SVM model.

## Discussion

In this study, we constructed a binary classification model to predict M/P_AUC_ using two models, ANN and SVM. Descriptors calculated specifically from the molecular structure of the compounds were used in the predictions. The performance of the models was within the acceptable range for both ANN and SVM. The ADMET Predictor^®^ is a software package that can rapidly predict ADMET (absorption, distribution, metabolism, excretion, and toxicity) properties based on molecular structures [34]. The predictive model is built using carefully selected descriptors and optimal learning algorithms based on actual measurements collected from public sources and academic literature. It shows excellent performance in predicting the aqueous solubility of chemicals [35], organic compounds [36], and the plasma protein binding of compounds in humans [37]. The ADMET Modeler™ module allows users to build QSAR/QSPR prediction models from chemical structures and measured datasets. These were also used to develop *in silico* models to identify androgen-active chemicals [38] and to build machine-learning models to predict chemotherapy-induced peripheral neuropathy [39]. The ADMET Modeler^TM^ is equipped with an early learning stop system to prevent overlearning, compensating for the shortcomings of ANNs, which are known to be prone to overlearning. The performance of the ANN model was checked using confidence analysis. A maximum uncertainty of 40–60% is recommended as the statistical stability of the model [40]. Therefore, the model we constructed with 57.5% uncertainty meets this requirement.

We focused on the AUC to evaluate the M/P ratio. In the data collected, M/P_AUC_ was obtained for only 43% of the 403 compounds with M/P ratio data. The problems with evaluating non-AUC M/P ratio with have been discussed in previous studies [41]. Furthermore, we excluded colostrum data and ultimately built our model with 129 compounds. While some of the previously reported models covered more than 300 compounds, curation allowed us to use a carefully selected dataset of compounds in this study. This is the first report of curation using these criteria for over 400 compounds.

In this study, the models were constructed to identify compounds with M/P_AUC_ greater than 1. Of the compounds with M/P_AUC_ greater than 1 collected from the original articles, none showed false negative results in either model. Specifically, the M/P_AUC_ ratios of 1.43 and 1.77 for mirtazapine and moxidectin [42, 43], respectively, were less than 1 by the ANN model and were false negative, while the SVM model correctly predicted their values greater than 1. The M/P_AUC_ ratios of 1.32 and 5.99 for fluvoxamine and N-monodesalkyldisopyramide [44, 45], respectively, were predicted to be less than 1 by the SVM model and were false negative, while the ANN model correctly predicted these values greater than 1. Therefore, we believe that a combined analysis of various models will also contribute to model reconstruction in the future and improvement in accuracy, although the results of prediction and reliability analyses for compounds near the M/P_AUC_=1 boundary value need to be carefully considered.

Although the ANN and SVM models had different contributing descriptors, both models were characterized by a high number of charge-based descriptors among the top 10 contributing descriptors. Charge-based descriptors with high contributions in the ANN model included EEM_XFc, Pi_AQc, Pi_FPl4, MinQ, and EEM_XFpl. Charge-based descriptors with high contribution rates in the SVM model included EEM_NFpl, EEM_Xfon, NPA_Q3, EEM_XFc, EEM_NFnp, and NPA_MinQ. Among them, EEM_XFc, EEM_XFp, EEM_NFpl, EEM_Xfon, and EEM_NFnp are the sigma Fukui indices, derivatives of the atomic partial charge relative to the total number of electrons. The sigma charges were provided as input to parameterize the two-dimensional deformation of the EEM based on Chaves' formalism [46] for the EEM kernel. Fukui (+) indicators like Pi_FPl4 are related to the Pi electron density in the lowest unoccupied molecular orbital (LUMO) [47]. Previously reported milk transfer prediction models suggested that electronic properties play an important role [18, 24, 48], consistent with the high contribution of electronic properties in this study. The "SaaaC" with the highest contribution in the SVM model was Atom-type Electropological State Indices. It is dominated by the E-state descriptor, an electrophysiological state indicator for the atom type, which is incrementally perturbed by the eigenstates of the atoms to which they are connected and weighted by the topological distance to each other [49]. In addition, "S+logD" simulated by ADMET Predictor also has a high contribution in the SVM model, confirming that fat solubility is also an important factor. S+logD indicates values related to pKa and log P. pKa and logP are important factors in the evaluation of milk transferability according to previously published models for predicting milk transfer [16, 17]. We performed a preliminary study using descriptors that can be added arbitrarily to the ADMET Modelar^TM^. The results confirmed that adding S+logD to the descriptors in the SVM model improved its accuracy. A similar trend was not observed in the ANN model. We plan to further examine how known factors related to milk transfer affect the construction of the QSAR/QSPR model.

The M/P_AUC_ prediction models developed in this study can be used to help evaluate the milk transfer potential of drugs, metabolites, impurities, and even enantiomers that have never been administered to lactating women. A case of child death due to morphine intoxication caused by breastfeeding from a mother who took codeine has been reported [50]. Although one factor in this case was that the mother was an ultra-rapid metabolizer of CYP2D6, an enzyme that metabolizes codeine, it is important to recognize that exposure to metabolites can affect the infant. Compounds like fluoxetine need to be evaluated for metabolites and racemates [51].

Since transporters such as BCRP have been shown to be involved in the milk transfer of drugs [52], it is important to know whether a compound is a substrate drug. If significant milk transfer is observed, contrary to the results predicted by pH partitioning theory, active transport may be involved. The ADMET Predictor^®^ has a module that predicts whether a compound can be a substrate for BCRP. This predictive model showed 85.9% concordance for the training set and 85.6% for the test set [40]. Thus, the QSAR model can predict the characteristics of drug milk transfer, including the involvement of transporters, which are difficult to predict based on the physicochemical properties alone. However, BCRP is not the only transporter whose expression increases in the mammary gland during lactation; there are also the sodium/iodide symporter and Organic Cation Transporter 1, and there may be other transporters that are not yet known [52]. If predictive modeling indicates that active transport may be involved in milk transfer of a target compound, more detailed clinical data is needed.

This study has several limitations, including data consistency for model building. We curated the reported data in order to build an accurate model. However, the M/P_AUC_ data were collected from various papers and thus the target patients and measurement methods were not standardized. In addition, M/P cannot be quantitatively evaluated in the classification model constructed in this study. Due to the ethical challenges in conducting large clinical trials in lactating women, much of the data must rely on case reports. We hope that further clinical data will be available in the future, as well as improvements in the *in silico* prediction accuracy. Furthermore, the compounds to which the ADMET predictor^®^ can be applied only include organic compounds consisting of boron, carbon, nitrogen, oxygen, sulfur, phosphorus, fluorine, chlorine, bromine, and iodine. Therefore, it is unsuitable for the prediction of compounds containing metals or polymers. However, the constructed predictive model can be applied to many drugs, and polymeric compounds are less likely to migrate into milk. For example, the milk: serum ratios of abatacept and tocilizumab have been reported to be 0.003–0.005 and 0.001–0.002, respectively [53, 54].

It is not appropriate to predict the advisability of breastfeeding during drug therapy solely on the basis of the M/P ratio; the child's drug intake and metabolic capacity should also be considered. A physiologically based pharmacokinetic model for predicting milk transfer of drugs has been constructed and is anticipated to be applied to some drugs [55, 56]. As new drugs are launched, medical professionals evaluate milk transfer based on the results of animal experiments, physicochemical properties of compounds, or past case reports, and consider drug therapy for lactating women as a time-consuming process. If *in silico* prediction can be used to evaluate the milk transfer properties of drugs quickly and easily, the possibility of achieving compatibility between drug therapy and breastfeeding can be increased. It is also necessary to analyze the prediction results obtained by machine learning not only with one model but a combination of models to avoid deterioration of the prediction accuracy [57]. We believe that the model developed in this study can assist in the evaluation of the milk transfer characteristics of compounds, especially those involving active transport by BCRP.

## Conclusions

The purpose of this study was to develop a model to predict whether the human M/P_AUC_ would exceed 1 for screening drugs transported actively in milk transfer. We built a milk transfer prediction model based on QSAR/QSPR using two methods, ANN and SVM. These two models showed satisfactory performance. Subsequently, in the process of building the predictive model, we confirmed the high contribution of the charge-based descriptor. The specific charge-based descriptor types and contribution rates, their relationship to active transport, and their effects on M/P ratios require further investigation but the charge properties indicated that these may be added to molecular weight, plasma protein binding, lipid solubility, and acid–base properties as factors to evaluate milk transfer of drugs.

Although further study is needed for the descriptors, we believe that the model constructed in this study can play an auxiliary role in evaluating the milk transferability of drugs.

## Supplementary Information

Below is the link to the electronic supplementary material.

Supplementary file1 (PDF 1213 KB)

## Data Availability

The datasets generated during and/or analyzed during the current study are available from the corresponding author on reasonable request.

## References

[1] Ladomenou F, Moschandreas J, Kafatos A, Tselentis Y, Galanakis E (2010). Protective effect of exclusive breastfeeding against infections during infancy: a prospective study. Arch Dis Child.

[2] Kramer MS, Aboud F, Mironova E, Vanilovich I, Platt RW, Matush L (2008). Breastfeeding and child cognitive development: new evidence from a large randomized trial. Arch Gen Psychiatry.

[3] Horta BL, de Lima NP (2019). Breastfeeding and Type 2 diabetes: systematic review and meta-analysis. Curr Diab Rep.

[4] Gunderson EP, Hurston SR, Ning X, Lo JC, Crites Y, Walton D (2015). Lactation and progression to type 2 diabetes mellitus after gestational diabetes mellitus: A prospective cohort study. Ann Intern Med.

[5] Schwarz EB, Ray RM, Stuebe AM, Allison MA, Ness RB, Freiberg MS (2009). Duration of lactation and risk factors for maternal cardiovascular disease. Obstet Gynecol.

[6] Weaver JM, Schofield TJ, Papp LM (2018). Breastfeeding duration predicts greater maternal sensitivity over the next decade. Dev Psychol.

[7] World Health Organization. Global strategy for infant and young child feeding; 2003. https://www.who.int/publications/i/item/9241562218. Accessed 18 Oct 2021.

[8] Ministry of Health, Labour and Welfare. Heisei. 27 nendo nyuyoji eiyo chosa kekka no gaiyo; 2015. https://www.mhlw.go.jp/file/06-Seisakujouhou-11900000-Koyoukintoujidoukateikyoku/0000134207.pdf. Accessed 18 Oct 2021 [Results of the 2015 infant nutrition survey].

[9] Ministry of Health, Labour and Welfare. The 2nd Study Group on the Insurance and Medical System for Expectant and Nursing Mothers. Current Status and Issues in the Medical Care of Pregnant and Nursing Women [Ninsanpu no shinryo no genjyo to kadai (in Japanese)]; 2019. https://www.mhlw.go.jp/content/12401000/000488877.pdf. Accessed 12 May 2022.

[10] Verstegen RHJ, Ito S (2019). Drugs in lactation. J Obstet Gynaecol Res.

[11] Ito N, Ito K, Ikebuchi Y, Toyoda Y, Takada T, Hisaka A (2015). Prediction of drug transfer into milk considering breast cancer resistance protein (BCRP)-mediated transport. Pharm Res.

[12] Verstegen RHJ, Anderson PO, Ito S (2020). Infant drug exposure via breast milk. Br J Clin Pharmacol.

[13] Ito S, Koren G (1994). A novel index for expressing exposure of the infant to drugs in breast milk. Br J Clin Pharmacol.

[14] Anderson PO, Momper JD (2020). Clinical lactation studies and the role of pharmacokinetic modeling and simulation in predicting drug exposures in breastfed infants. J Pharmacokinet Pharmacodyn.

[15] Wilson JT, Brown RD, Cherek DR, Dailey JW, Hilman B, Jobe PC (1980). Drug excretion in human breast milk: principles, pharmacokinetics and projected consequences. Clin Pharmacokinet.

[16] Begg EJ, Atkinson HC (1993). Modelling of the passage of drugs into milk. Pharmacol Ther.

[17] Meskin MS, Lien EJ (1985). QSAR analysis of drug excretion into human breast milk. J Clin Hosp Pharm.

[18] Agatonovic-Kustrin S, Tucker IG, Zecevic M, Zivanovic LJ (2000). Prediction of drug transfer into human milk from theoretically derived descriptors. Anal Chim Acta.

[19] Katritzky AR, Dobchev DA, Hür E, Fara DC, Karelson M (2005). QSAR treatment of drugs transfer into human breast milk. Bioorg Med Chem.

[20] Abraham MH, Gil-Lostes J, Fatemi M (2009). Prediction of milk/plasma concentration ratios of drugs and environmental pollutants. Eur J Med Chem.

[21] Agatonovic-Kustrin S, Morton DW, Celebic D (2013). QSAR: an *in silico* approach for predicting the partitioning of pesticides into breast milk. Comb Chem High Throughput Screen.

[22] Kar S, Roy K (2013). Prediction of Milk/Plasma Concentration Ratios of Drugs and Environmental Pollutants Using *In Silico* Tools: Classification and Regression Based QSARs and Pharmacophore Mapping. Mol Inform.

[23] Wanat K, Khakimov B, Brzezińska E (2020). Comparison of statistical methods for predicting penetration capacity of drugs into human breast milk using physicochemical, pharmacokinetic and chromatographic descriptors. SAR QSAR Environ Res.

[24] Zhao C, Zhang H, Zhang X, Zhang R, Luan F, Liu M (2006). Prediction of milk/plasma drug concentration (M/P) ratio using support vector machine (SVM) method. Pharm Res.

[25] Fatemi MH, Ghorbanzad'e M (2010). Classification of drugs according to their milk/plasma concentration ratio. Eur J Med Chem.

[26] Somogyi A, Gugler R (1979). Cimetidine excretion into breast milk. Br J Clin Pharmacol.

[27] Shyu WC, Shah VR, Campbell DA, Venitz J, Jaganathan V, Pittman KA (1992). Excretion of cefprozil into human breast milk. Antimicrob Agents Chemother.

[28] Hale TW. Medications & Mothers' Milk. Springer Publishing company; 2019.

[29] Briggs GG. Drugs in pregnancy and lactation : a reference guide to fetal and neonatal risk. 11th ed. Lippincott Williams & Wilkins; 2017.

[30] Drugs and Lactation Database (LactMed); 2006. https://www.ncbi.nlm.nih.gov/books/NBK501922/. Accessed 17 Sep 2019.

[31] PubChem. https://pubchem.ncbi.nlm.nih.gov/. Accessed 13 Jul 2020.

[32] Kohonen T (1982). Self-organized formation of topologically correct feature maps. Biological Cybernetics volume.

[33] Leardi R, Boggia R, Terrile M (1992). Genetic algorithms as a strategy for feature selection. J Chemometrics.

[34] Simulation Plus Website. https://www.simulations-plus.com/ Accessed 19 Nov 2022.

[35] Hewitt M, Cronin MT, Enoch SJ, Madden JC, Roberts DW, Dearden JC (2009). *In silico* prediction of aqueous solubility: the solubility challenge. J Chem Inf Model.

[36] Cappelli CI, Manganelli S, Lombardo A, Gissi A, Benfenati E (2013). Validation of quantitative structure-activity relationship models to predict water-solubility of organic compounds. Sci Total Environ.

[37] Yun YE, Tornero-Velez R, Purucker ST, Chang DT, Edginton AN (2021). Evaluation of Quantitative Structure Property Relationship Algorithms for Predicting Plasma Protein Binding in Humans. Comput Toxicol..

[38] Manganelli S, Roncaglioni A, Mansouri K, Judson RS, Benfenati E, Manganaro A (2019). Development, validation and integration of *in silico* models to identify androgen active chemicals. Chemosphere.

[39] Bloomingdale P, Mager DE (2019). Machine Learning Models for the Prediction of Chemotherapy-Induced Peripheral Neuropathy. Pharm Res.

[40] Simulation Plus I. ADMET Predictor X.3 Manual. 2021.

[41] Wilson JT, Brown RD, Hinson JL, Dailey JW (1985). Pharmacokinetic pitfalls in the estimation of the breast milk/plasma ratio for drugs. Annu Rev Pharmacol Toxicol.

[42] Kristensen JH, Ilett KF, Rampono J, Kohan R, Hackett LP (2007). Transfer of the antidepressant mirtazapine into breast milk. Br J Clin Pharmacol.

[43] Korth-Bradley JM, Parks V, Chalon S, Gourley I, Matschke K, Gossart S (2011). Excretion of moxidectin into breast milk and pharmacokinetics in healthy lactating women. Antimicrob Agents Chemother.

[44] Hägg S, Granberg K, Carleborg L (2000). Excretion of fluvoxamine into breast milk. Br J Clin Pharmacol.

[45] Ellsworth AJ, Horn JR, Raisys VA, Miyagawa LA, Bell JL (1989). Disopyramide and N-monodesalkyl disopyramide in serum and breast milk. DICP.

[46] Chaves J, Barroso JM, Bultinck P, Carbó-Dorca R (2006). Toward an alternative hardness kernel matrix structure in the Electronegativity Equalization Method (EEM). J Chem Inf Model.

[47] Anderson JSM, Melin J, Ayers PW (2007). Conceptual Density-Functional Theory for General Chemical Reactions, Including Those That Are Neither Charge- nor Frontier-Orbital-Controlled. 1. Theory and Derivation of a General-Purpose Reactivity Indicator. J Chem Theor Comput..

[48] Agatonovic-Kustrin S, Ling LH, Tham SY, Alany RG (2002). Molecular descriptors that influence the amount of drugs transfer into human breast milk. J Pharm Biomed Anal.

[49] Hall LH, Mohney B, Kier LB (1991). The electrotopological state: structure information at the atomic level for molecular graphs. J Chem Inf Comput Sci.

[50] Koren G, Cairns J, Chitayat D, Gaedigk A, Leeder SJ (2006). Pharmacogenetics of morphine poisoning in a breastfed neonate of a codeine-prescribed mother. Lancet.

[51] Anderson PO, Sauberan JB (2016). Modeling drug passage into human milk. Clin Pharmacol Ther.

[52] García-Lino AM, Álvarez-Fernández I, Blanco-Paniagua E, Merino G, Álvarez AI. Transporters in the Mammary Gland-Contribution to Presence of Nutrients and Drugs into Milk. Nutrients. 2019;11. 10.3390/nu11102372. 10.3390/nu11102372 PMC6836069 31590349

[53] Saito J, Yakuwa N, Takai C, Kaneko K, Goto M, Nakajima K (2019). Abatacept concentrations in maternal serum and breast milk during breastfeeding and an infant safety assessment: a case study. Rheumatol (Oxf Engl).

[54] Saito J, Yakuwa N, Takai C, Nakajima K, Kaneko K, Goto M (2018). Tocilizumab concentrations in maternal serum and breast milk during breastfeeding and a safety assessment in infants: a case study. Rheumatol (Oxf Engl).

[55] Job KM, Dallmann A, Parry S, Saade G, Haas DM, Hughes B (2022). Development of a Generic Physiologically-Based Pharmacokinetic Model for Lactation and Prediction of Maternal and Infant Exposure to Ondansetron via Breast Milk. Clin Pharmacol Ther.

[56] Abduljalil K, Pansari A, Ning J, Jamei M (2021). Prediction of drug concentrations in milk during breastfeeding, integrating predictive algorithms within a physiologically-based pharmacokinetic model. CPT Pharmacometrics Syst Pharmacol.

[57] Kiryu Y (2022). Medical Big Data Analysis Using Machine Learning Algorithms in the Field of Clinical Pharmacy. Yakugaku Zasshi.

