# Supplementary material for: Prediction model for milk transfer of drugs by primarily evaluating the area under the curve using QSAR/QSPR
Source: Pharm Res. 2023 Jan 31;40(3):711–9. doi: 10.1007/s11095-023-03477-1 (PMC10036427; doi:10.1007/s11095-023-03477-1)
Supplement: Supplementary file 1 — Supplementary file1 (PDF 1213 KB) [file 11095_2023_3477_MOESM1_ESM.pdf]

Online Resource List of compounds used in model construction

Compound name	Data set affiliation		Observed		Predicted M/P_{AUC} classification		Ref.
	ANN model	SVM model	M/P_{AUC}	M/P_{AUC} classification	ANN model (confidence)	SVM model	
Acetaminophen	Tr	V	1.6[a]	H	H (95%)	H	(1)
Acyclovir	Tr	Tr	2.69[a]	H	H (73%)	H	(2)
Alphamethyldopa	Tr	Tr	0.22	L	L (98%)	L	(3)
Alprazolam	Te	Tr	0.36	L	L (60%)	L	(4)
Amitriptyline	V	Tr	0.83[b]	L	L (98%)	L	(5)
Apixaban	V	Tr	2.61	H	H (87%)	H	(6)
Atenolol	Te	Tr	5.7	H	H (53%)	H	(7)
Baclofen	Tr	Tr	0.72[b]	L	L (98%)	L	(8)
Budesonide	Te	Te	0.5	L	L (98%)	L	(9)
Buprenorphine	Tr	Tr	1.73	H	H (95%)	H	(10)
Caffeine	Tr	Tr	0.7	L	L (98%)	L	(11)
Candesartan	Tr	Tr	0.02	L	L (77%)	L	(12)
Cefprozil	Tr	Te	0.6	L	L (98%)	L	(13)
Celecoxib	Tr	Tr	0.18	L	L (81%)	L	(14)
Chlorprothixene	V	Tr	1.31	H	H (77%)	H	(15)
Chlorprothixene sulphoxide	Tr	V	0.67[b]	L	L (94%)	L	(15)
Citalopram	V	V	1.8	H	H (95%)	H	(16)
Clarithromycin	V	V	0.25	L	L (88%)	L	(17)
Codeine	Tr	Tr	2.16	H	H (62%)	H	(18)
Colchicine	Tr	Tr	0.52[a]	L	L (98%)	L	(19)
Cotinine	Tr	Tr	1.2	H	H (95%)	H	(20)
Dapivirine	Tr	Tr	1.8	H	H (77%)	H	(21)
Dehydronitrendipine	Tr	Te	0.6	L	L (98%)	L	(22)
Demethylcitalopram	Te	Te	1.8	H	H (95%)	H	(16)
Descarboethoxyloratadine	Te	Tr	0.8	L	L (74%)	L	(23)
Desmethylmirtazapine	Tr	Tr	0.45[a]	L	L (98%)	L	(24)
Dexamphetamine	Tr	Tr	2.2	H	H (95%)	H	(25)
Digoxin	Tr	Tr	0.85	L	L (94%)	L	(26)
Diltiazem	Tr	Tr	0.99[a]	L	L (98%)	L	(27)
Disopyramide	Tr	Tr	0.41	L	L (69%)	L	(28)
Doxazosin	Tr	Tr	0.1	L	L (98%)	L	(29)
Doxorubicin	Tr	Tr	1.19	H	H (95%)	H	(30)
Doxorubicinol	V	V	9.71	H	H (95%)	H	(30)
Drospirenone	Tr	Tr	0.18	L	L (91%)	L	(31)
Duloxetine	Tr	Tr	0.27	L	L (98%)	L	(32)

Efavirenz	Tr	Tr	0.84[a]	L	L (60%)	L	(33)
Emtricitabine	Tr	Tr	3.01	H	H (87%)	H	(34)
Escitalopram	Tr	Tr	2.2	H	H (95%)	H	(35)
Ethanol	Tr	Te	0.99[a]	L	L (60%)	L	(36)
Etravirine	V	V	3.47[b]	H	H (59%)	H	(37)
Fexofenadine	Tr	Tr	0.21	L	L (98%)	L	(38)
Fluconazole	Tr	Te	0.77[b]	L	H (62%)	L	(39)
Fluoxetine	V	V	0.52	L	L (88%)	L	(40)
Flurbiprofen	Tr	Tr	0.02	L	L (94%)	L	(41)
Fluvoxamine	Te	Te	1.32[a]	H	H (62%)	L	(42)
Hydrochlorothiazide	Tr	V	0.46[a]	L	L (77%)	L	(43)
14-Hidroxyclarithromycin	Tr	Tr	0.66	L	L (88%)	L	(17)
Hydroxynefazodone	V	V	0.07	L	L (94%)	L	(44)
Imatinib Mesylate	Tr	Tr	0.37[a]	L	L (70%)	L	(45)
Labetalol	Tr	V	0.8	L	L (76%)	L	(46)
Lamivudine	V	V	1.31	H	H (95%)	H	(47)
Levodopa	V	V	0.32	L	L (98%)	L	(48)
Levonorgestrel	V	V	0.26	L	L (79%)	L	(49)
Lopinavir	Tr	Tr	0.19	L	L (98%)	L	(50)
Loratadine	Tr	Tr	1.2	H	H (87%)	H	(23)
m-Chlorophenylpiperazine	Tr	Tr	0.19	L	H (82%)	L	(44)
Metformin	Tr	Te	0.4	L	L (64%)	L	(51)
(R)-Methadone	Tr	Tr	0.47	L	L (88%)	L	(52)
(S)-Methadone	V	V	0.27	L	L (88%)	L	(52)
Methotrexate	Te	Tr	0.04[a]	L	L (94%)	L	(53)
Metoprolol	Tr	Tr	2.4	H	H (82%)	H	(54)
Minoxidil	V	Tr	0.75[b]	L	H (47%)	L	(55)
Minoxidil Glucuronide	Tr	V	0.12[b]	L	L (98%)	L	(55)
Mirtazapine	Te	Tr	1.43[a]	H	L (86%)	H	(24)
Monomethyl 2,6-dimethyl-4-(2-nitrophenyl)-3,5-pyridinedicarboxylate	V	Te	0.88[a]	L	L (98%)	L	(56)
Morphine	Te	Te	2.45	H	H (65%)	H	(57)
Moxidectin	Tr	Tr	1.77	H	L (83%)	H	(58)
Nadolol	Tr	Tr	4.6	H	H (95%)	H	(59)
N-Desalkyl-2-oxoquazepam	V	Te	0.09	L	L (98%)	L	(60)
N-Desmethyl Imatinib	V	V	0.71[a]	L	L (70%)	L	(45)
N-Desmethylsertraline	Tr	V	1.64	H	H (70%)	H	(61)
Nefazodone	Tr	Tr	0.27	L	L (94%)	L	(44)
Nevirapine	V	V	0.95	L	L (98%)	L	(62)
Nicotine	Tr	Tr	1.5	H	H (95%)	H	(20)

Nifedipine	V	V	0.74[a]	L	L (98%)	L	(56)
Nimodipine	Te	Te	0.11	L	L (86%)	L	(63)
Nitrendipine	Tr	Tr	0.82	L	L (98%)	L	(22)
Nitrofurantoin	Tr	Tr	6.2	H	H (69%)	H	(64)
N-Monodesalkyldisopyramide	V	V	5.99	H	H (73%)	L	(65)
Norbuprenorphine	V	V	0.73	L	H (56%)	L	(10)
Norfluoxetine	Tr	Tr	0.42	L	L (81%)	L	(40)
Nortriptyline	V	Tr	0.65[b]	L	L (98%)	L	(5)
Norverapamil	Tr	Tr	0.16	L	L (98%)	L	(66)
Noscapine	V	V	0.34	L	L (98%)	L	(67)
Olanzapine	Tr	Tr	0.38	L	L (76%)	L	(68)
2-Oxoquazepam	Tr	Tr	2.01	H	H (47%)	H	(60)
Paliperidone	Tr	Tr	0.24	L	L (98%)	L	(69)
Pantoprazole	V	Tr	0.03	L	L (98%)	L	(70)
Paraxanthine	Tr	Tr	0.52	L	L (98%)	L	(11)
Penicillin V	Tr	Tr	0.15	L	L (98%)	L	(71)
Phenacetin	V	Tr	0.67	L	L (65%)	L	(18)
Pilsicainide	Tr	Tr	1.75	H	H (95%)	H	(28)
Praziquantel	V	V	0.35	L	H (45%)	L	(72)
Prednisolone	Tr	Tr	0.03	L	L (98%)	L	(73)
Predonisone	V	V	0.55	L	L (98%)	L	(73)
Pregabalin	V	V	0.76	L	L (98%)	L	(74)
Propafenone	V	Tr	0.25	L	L (86%)	L	(28)
Propranolol	Tr	V	0.32[b]	L	L (64%)	L	(75)
Propylthiouracil	Tr	Tr	0.09[a]	L	L (91%)	L	(76)
Pseudoephedrine	V	V	2.48	H	H (95%)	H	(77)
Quazepam	Te	V	4.18	H	H (45%)	H	(60)
Quetiapine	V	V	0.29	L	L (88%)	L	(78)
Quinapril	V	Tr	0.12	L	L (98%)	L	(79)
Ranitidine	Tr	Tr	9.83[b]	H	H (56%)	H	(80)
Reboxetine	Tr	Tr	0.06	L	L (76%)	L	(81)
Risperidone	V	V	0.42	L	L (98%)	L	(69)
Ritonavir	V	V	0.2	L	L (98%)	L	(50)
Rivaroxaban	Tr	V	0.27	L	L (88%)	L	(6)
Rofecoxib	Tr	V	0.25	L	L (98%)	L	(82)
Rosaramicin	Tr	V	0.12	L	L (98%)	L	(83)
Roxithromycin	Te	Te	0.03	L	L (83%)	L	(84)
Saccharin	Tr	Tr	0.72	L	L (91%)	L	(85)
Salicylate	V	V	0.05	L	L (88%)	L	(18)
Sertraline	V	V	1.93	H	H (95%)	H	(61)

Sumatriptan	Tr	Tr	4.9	H	H (42%)	H	(86)
Suprofen	Tr	Tr	0.01	L	L (98%)	L	(87)
Tacrolimus	Tr	Tr	0.13	L	L (94%)	L	(88)
Tenofovir	Tr	Tr	0.02	L	L (67%)	L	(34)
Terbutaline	Tr	Tr	1.04	H	H (95%)	H	(89)
Theobromine	V	V	0.82	L	L (98%)	L	(11)
Theophylline	Te	Te	0.57	L	L (98%)	L	(11)
Thiopental Sodium	Tr	V	0.44[b]	L	L (81%)	L	(90)
Tolmetin	V	V	0.01	L	L (98%)	L	(91)
Trazodone	Te	Te	0.14	L	L (81%)	L	(92)
Triazoledione	Tr	Te	0.02	L	L (98%)	L	(44)
Triprolidine	Tr	V	0.53	L	L (88%)	L	(77)
Verapamil	V	V	0.6	L	L (98%)	L	(66)
Zaleplon	Tr	Tr	0.5	L	L (94%)	L	(93)
Zidovudine	Tr	Tr	1.35	H	H (65%)	H	(50)

a : Value calculated by reproducing the graph using DegitizeIt®

b : Value calculated using the trapezoidal method

H＝High (M/P_{AUC}≥1), L＝low (M/P_{AUC}＜1)

Tr: Training set, V: Verify set, Te: Test set

ANN; Artificial Neural Network, SVM; Support Vector Machine

Reference

1. Notarianni LJ, Oldham HG, Bennett PN. Passage of paracetamol into breast milk and its subsequent metabolism by the neonate. Br J Clin Pharmacol. 1987;24(1):63-7. https//doi.org/10.1111/j.1365-2125.1987.tb03137.x.
2. Bork K, Benes P. Concentration and kinetic studies of intravenous acyclovir in serum and breast milk of a patient with eczema herpeticum. J Am Acad Dermatol. 1995;32(6):1053-5. https//doi.org/ 10.1016/0190-9622(95)91362-9.
3. White WB, Andreoli JW, Cohn RD. Alpha-methyldopa disposition in mothers with hypertension and in their breast-fed infants. Clin Pharmacol Ther. 1985;37(4):387-90. https//doi.org/ 10.1038/clpt.1985.59.
4. Oo CY, Kuhn RJ, Desai N, Wright CE, McNamara PJ. Pharmacokinetics in lactating women: prediction of alprazolam transfer into milk. Br J Clin Pharmacol. 1995;40(3):231-6. https//doi.org/ 10.1111/j.1365-2125.1995.tb05778.x.
5. Pittard WB, 3rd, O'Neal W, Jr. Amitriptyline excretion in human milk. J Clin Psychopharmacol. 1986;6(6):383-4. https//doi.org/10.1097/00004714-198612000-00024.
6. Zhao Y, Arya R, Couchman L, Patel JP. Are apixaban and rivaroxaban distributed into human breast milk to clinically relevant concentrations? Blood. 2020;136(15):1783-5.

https//doi.org/10.1182/blood.2020006231.

7. Eyal S, Kim JD, Anderson GD, Buchanan ML, Brateng DA, Carr D, et al. Atenolol pharmacokinetics and excretion in breast milk during the first 6 to 8 months postpartum. J Clin Pharmacol. 2010;50(11):1301-9. https//doi.org/10.1177/0091270009358708.
8. Eriksson G, Swahn CG. Concentrations of baclofen in serum and breast milk from a lactating woman. Scand J Clin Lab Invest. 1981;41(2):185-7. https//doi.org/10.3109/00365518109092032.
9. Falt A, Bengtsson T, Kennedy BM, Gyllenberg A, Lindberg B, Thorsson L, et al. Exposure of infants to budesonide through breast milk of asthmatic mothers. J Allergy Clin Immunol. 2007;120(4):798-802. https//doi.org/10.1016/j.jaci.2007.07.023.
10. Lindemalm S, Nydert P, Svensson JO, Stahle L, Sarman I. Transfer of buprenorphine into breast milk and calculation of infant drug dose. J Hum Lact. 2009;25(2):199-205. https//doi.org/ 10.1177/0890334408328295.
11. Oo CY, Burgio DE, Kuhn RC, Desai N, McNamara PJ. Pharmacokinetics of caffeine and its demethylated metabolites in lactation: predictions of milk to serum concentration ratios. Pharm Res. 1995;12(2):313-6. https//doi.org/10.1023/a:1016207832591.
12. Coberger ED, Jensen BP, Dalrymple JM. Transfer of Candesartan Into Human Breast Milk. Obstet Gynecol. 2019;134(3):481-4. https//doi.org/10.1097/aog.0000000000003446.
13. Shyu WC, Shah VR, Campbell DA, Venitz J, Jaganathan V, Pittman KA, et al. Excretion of cefprozil into human breast milk. Antimicrob Agents Chemother. 1992;36(5):938-41. https//doi.org/10.1128/aac.36.5.938.
14. Gardiner SJ, Doogue MP, Zhang M, Begg EJ. Quantification of infant exposure to celecoxib through breast milk. Br J Clin Pharmacol. 2006;61(1):101-4. https//doi.org/10.1111/j.1365-2125.2005.02520.x.
15. Matheson I, Evang A, Overø KF, Syversen G. Presence of chlorprothixene and its metabolites in breast milk. Eur J Clin Pharmacol. 1984;27(5):611-3. https//doi.org/10.1007/bf00556901.
16. Rampono J, Kristensen JH, Hackett LP, Paech M, Kohan R, Ilett KF. Citalopram and demethylcitalopram in human milk; distribution, excretion and effects in breast fed infants. Br J Clin Pharmacol. 2000;50(3):263-8. https//doi.org/10.1046/j.1365-2125.2000.00253.x.
17. Sedlmayr T, Peters F, Raasch W, Kees F. Clarithromycin, a new macrolide antibiotic. Effectiveness in puerperal infections and pharmacokinetics in breast milk. Geburtshilfe Frauenheilkd. 1993;53(7):488-91. https//doi.org/10.1055/s-2007-1022919.
18. Findlay JW, DeAngelis RL, Kearney MF, Welch RM, Findlay JM. Analgesic drugs in breast milk and plasma. Clin Pharmacol Ther. 1981;29(5):625-33. https//doi.org/10.1038/clpt.1981.87.
19. Ben-Chetrit E, Scherrmann JM, Levy M. Colchicine in breast milk of patients with familial Mediterranean fever. Arthritis Rheum. 1996;39(7):1213-7. https//doi.org/10.1002/art.1780390721.
20. Ilett KF, Hale TW, Page-Sharp M, Kristensen JH, Kohan R, Hackett LP. Use of nicotine patches in breast-feeding mothers: transfer of nicotine and cotinine into human milk. Clin Pharmacol Ther. 2003;74(6):516-24. https//doi.org/10.1016/j.clpt.2003.08.003.
21. Noguchi LM, Hoesley C, Kelly C, Scheckter R, Bunge K, Nel A, et al. Pharmacokinetics of Dapivirine Transfer into Blood Plasma, Breast Milk, and Cervicovaginal Fluid of Lactating Women Using the Dapivirine Vaginal Ring. Antimicrob Agents Chemother. 2019;63(3). doi: 10.1128/aac.01930-18.

22. White WB, Yeh SC, Krol GJ. Nitrendipine in human plasma and breast milk. Eur J Clin Pharmacol. 1989;36(5):531-4. https//doi.org/10.1007/BF00558082.
23. Hilbert J, Radwanski E, Affrime MB, Perentesis G, Symchowicz S, Zampaglione N. Excretion of loratadine in human breast milk. J Clin Pharmacol. 1988;28(3):234-9. https//doi.org/10.1002/j.1552-4604.1988.tb03138.x.
24. Kristensen JH, Ilett KF, Rampono J, Kohan R, Hackett LP. Transfer of the antidepressant mirtazapine into breast milk. Br J Clin Pharmacol. 2007;63(3):322-7. https//doi.org/10.1111/j.1365-2125.2006.02773.x.
25. Ilett KF, Hackett LP, Kristensen JH, Kohan R. Transfer of dexamphetamine into breast milk during treatment for attention deficit hyperactivity disorder. Br J Clin Pharmacol. 2007;63(3):371-5. https//doi.org/ 10.1111/j.1365-2125.2006.02767.x.
26. Loughnan PM. Digoxin excretion in human breast milk. J Pediatr. 1978;92(6):1019-20. https//doi.org/ 10.1016/s0022-3476(78)80391-6.
27. Okada M, Inoue H, Nakamura Y, Kishimoto M, Suzuki T. Excretion of diltiazem in human milk. N Engl J Med. 1985;312(15):992-3. https//doi.org/10.1056/nejm198504113121516.
28. Wakaumi M, Tsuruoka S, Sakamoto K, Shiga T, Fujimura A. Pilsicainide in breast milk from a mother: comparison with disopyramide and propafenone. Br J Clin Pharmacol. 2005;59(1):120-2. https//doi.org/ 10.1111/j.1365-2125.2004.02219.x.
29. Jensen BP, Dalrymple JM, Begg EJ. Transfer of doxazosin into breast milk. J Hum Lact. 2013;29(2):150-3. https//doi.org/10.1177/0890334412473203.
30. Egan PC, Costanza ME, Dodion P, Egorin MJ, Bachur NR. Doxorubicin and cisplatin excretion into human milk. Cancer Treat Rep. 1985;69(12):1387-89.
31. Melka D, Kask K, Colli E, Regidor PA. A single-arm study to evaluate the transfer of drospirenone to breast milk after reaching steady state, following oral administration of 4 mg drospirenone in healthy lactating female volunteers. Womens Health (Lond). 2020;16:1745506520957192. https//doi.org/ 10.1177/1745506520957192.
32. Lobo ED, Loghin C, Knadler MP, Quinlan T, Zhang L, Chappell J, et al. Pharmacokinetics of duloxetine in breast milk and plasma of healthy postpartum women. Clin Pharmacokinet. 2008;47(2):103-9. https//doi.org/10.2165/00003088-200847020-00003.
33. Olagunju A, Bolaji OO, Amara A, Waitt C, Else L, Soyinka J, et al. Development, validation and clinical application of a novel method for the quantification of efavirenz in dried breast milk spots using LC-MS/MS. J Antimicrob Chemother. 2015;70(2):555-61. https//doi.org/10.1093/jac/dku420.
34. Waitt C, Olagunju A, Nakalema S, Kyohaire I, Owen A, Lamorde M, et al. Plasma and breast milk pharmacokinetics of emtricitabine, tenofovir and lamivudine using dried blood and breast milk spots in nursing African mother-infant pairs. J Antimicrob Chemother. 2018;73(4):1013-9. https//doi.org/ 10.1093/jac/dkx507.
35. Rampono J, Hackett LP, Kristensen JH, Kohan R, Page-Sharp M, Ilett KF. Transfer of escitalopram and its metabolite demethylescitalopram into breastmilk. Br J Clin Pharmacol. 2006;62(3):316-22. https//doi.org/ 10.1111/j.1365-2125.2006.02659.x.
36. Chien YC, Liu JF, Huang YJ, Hsu CS, Chao JC. Alcohol levels in Chinese lactating mothers after

consumption of alcoholic diet during postpartum "doing-the-month" ritual. Alcohol. 2005;37(3):143-50. https//doi.org/10.1016/j.alcohol.2006.02.001.

37. Spencer LY, Liu S, Wang C-H, Neely M, Louie S, Kovacs A. Intensive etravirine PK and HIV-1 viral load in breast milk and plasma in HIV+ women receiving HAART. 2014;22:466.
38. Lucas BD, Jr., Purdy CY, Scarim SK, Benjamin S, Abel SR, Hilleman DE. Terfenadine pharmacokinetics in breast milk in lactating women. Clin Pharmacol Ther. 1995;57(4):398-402. https//doi.org/ 10.1016/0009-9236(95)90208-2.
39. Force RW. Fluconazole concentrations in breast milk. Pediatr Infect Dis J. 1995;14(3):235-6. https//doi.org/ 10.1097/00006454-199503000-00012.
40. Kristensen JH, Ilett KF, Hackett LP, Yapp P, Paech M, Begg EJ. Distribution and excretion of fluoxetine and norfluoxetine in human milk. Br J Clin Pharmacol. 1999;48(4):521-7. https//doi.org/10.1046/j.1365-2125.1999.00040.x.
41. Cox SR, Forbes KK. Excretion of flurbiprofen into breast milk. Pharmacotherapy. 1987;7(6):211-5. https//doi.org/10.1002/j.1875-9114.1987.tb03527.x.
42. Hägg S, Granberg K, Carleborg L. Excretion of fluvoxamine into breast milk. Br J Clin Pharmacol. 2000;49(3):286-8. https//doi.org/10.1046/j.1365-2125.2000.00142-3.x.
43. Miller ME, Cohn RD, Burghart PH. Hydrochlorothiazide disposition in a mother and her breast-fed infant. J Pediatr. 1982;101(5):789-91. https//doi.org/10.1016/s0022-3476(82)80323-5.
44. Yapp P, Ilett KF, Kristensen JH, Hackett LP, Paech MJ, Rampono J. Drowsiness and poor feeding in a breast-fed infant: association with nefazodone and its metabolites. Ann Pharmacother. 2000;34(11):1269-72. https//doi.org/10.1345/aph.10120.
45. Gambacorti-Passerini CB, Tornaghi L, Marangon E, Franceschino A, Pogliani EM, D'Incalci M, et al. Imatinib concentrations in human milk. Blood. 2007;109(4):1790. https//doi.org/10.1182/blood-2006-08-039545.
46. Lunell NO, Kulas J, Rane A. Transfer of labetalol into amniotic fluid and breast milk in lactating women. Eur J Clin Pharmacol. 1985;28(5):597-9. https//doi.org/10.1007/bf00544073.
47. Waitt C, Diliiy Penchala S, Olagunju A, Amara A, Else L, Lamorde M, et al. Development, validation and clinical application of a method for the simultaneous quantification of lamivudine, emtricitabine and tenofovir in dried blood and dried breast milk spots using LC-MS/MS. J Chromatogr B Analyt Technol Biomed Life Sci. 2017;1060:300-7. https//doi.org/10.1016/j.jchromb.2017.06.012.
48. Thulin PC, Woodward WR, Carter JH, Nutt JG. Levodopa in human breast milk: clinical implications. Neurology. 1998;50(6):1920-1. https//doi.org/10.1212/wnl.50.6.1920-a.
49. Gainer E, Massai R, Lillo S, Reyes V, Forcelledo ML, Caviedes R, et al. Levonorgestrel pharmacokinetics in plasma and milk of lactating women who take 1.5 mg for emergency contraception. Hum Reprod. 2007;22(6):1578-84. https//doi.org/10.1093/humrep/dem034.
50. Corbett AH, Kayira D, White NR, Davis NL, Kourtis AP, Chasela C, et al. Antiretroviral pharmacokinetics in mothers and breastfeeding infants from 6 to 24 weeks post-partum: results of the BAN Study. Antivir Ther. 2014;19(6):587-95. https//doi.org/10.3851/imp2739.
51. Eyal S, Easterling TR, Carr D, Umans JG, Miodovnik M, Hankins GD, et al. Pharmacokinetics of metformin during pregnancy. Drug Metab Dispos. 2010;38(5):833-40.

https//doi.org/10.1124/dmd.109.031245.

52. Begg EJ, Malpas TJ, Hackett LP, Ilett KF. Distribution of R- and S-methadone into human milk during multiple, medium to high oral dosing. Br J Clin Pharmacol. 2001;52(6):681-5. https//doi.org/10.1046/j.0306-5251.2001.01506.x.
53. Johns DG, Rutherford LD, Leighton PC, Vogel CL. Secretion of methotrexate into human milk. Am J Obstet Gynecol. 1972;112(7):978-80. https//doi.org/10.1016/0002-9378(72)90824-1.
54. Ryu RJ, Eyal S, Easterling TR, Caritis SN, Venkataraman R, Hankins G, et al. Pharmacokinetics of metoprolol during pregnancy and lactation. J Clin Pharmacol. 2016;56(5):581-9. https//doi.org/10.1002/jcph.631.
55. Valdivieso A, Valdés G, Spiro TE, Westerman RL. Minoxidil in breast milk. Ann Intern Med. 1985;102(1):135. https//doi.org/10.7326/0003-4819-102-1-135_1.
56. Penny WJ, Lewis MJ. Nifedipine is excreted in human milk. Eur J Clin Pharmacol. 1989;36(4):427-8. https//doi.org/10.1007/bf00558309.
57. Feilberg VL, Rosenborg D, Broen Christensen C, Mogensen JV. Excretion of morphine in human breast milk. Acta Anaesthesiol Scand. 1989;33(5):426-8. https//doi.org/10.1111/j.1399-6576.1989.tb02938.x.
58. Korth-Bradley JM, Parks V, Chalon S, Gourley I, Matschke K, Gossart S, et al. Excretion of moxidectin into breast milk and pharmacokinetics in healthy lactating women. Antimicrob Agents Chemother. 2011;55(11):5200-4. https//doi.org/10.1128/aac.00311-11.
59. Devlin RG, Duchin KL, Fleiss PM. Nadolol in human serum and breast milk. Br J Clin Pharmacol. 1981;12(3):393-6. https//doi.org/10.1111/j.1365-2125.1981.tb01232.x.
60. Hilbert JM, Gural RP, Symchowicz S, Zampaglione N. Excretion of quazepam into human breast milk. J Clin Pharmacol. 1984;24(10):457-62. https//doi.org/10.1002/j.1552-4604.1984.tb01819.x.
61. Kristensen JH, Ilett KF, Dusci LJ, Hackett LP, Yapp P, Wojnar-Horton RE, et al. Distribution and excretion of sertraline and N-desmethylsertraline in human milk. Br J Clin Pharmacol. 1998;45(5):453-7. https//doi.org/10.1046/j.1365-2125.1998.00705.x.
62. Olagunju A, Khoo S, Owen A. Pharmacogenetics of nevirapine excretion into breast milk and infants' exposure through breast milk versus postexposure prophylaxis. Pharmacogenomics. 2016;17(8):891-906. https//doi.org/10.2217/pgs-2015-0016.
63. Carcas AJ, Abad-Santos F, de Rosendo JM, Frias J. Nimodipine transfer into human breast milk and cerebrospinal fluid. Ann Pharmacother. 1996;30(2):148-50. https//doi.org/10.1177/106002809603000208.
64. Gerk PM, Kuhn RJ, Desai NS, McNamara PJ. Active transport of nitrofurantoin into human milk. Pharmacotherapy. 2001;21(6):669-75. https//doi.org/10.1592/phco.21.7.669.34574.
65. Ellsworth AJ, Horn JR, Raisys VA, Miyagawa LA, Bell JL. Disopyramide and N-monodesalkyl disopyramide in serum and breast milk. Dicp. 1989;23(1):56-7. https//doi.org/10.1177/106002808902300112.
66. Anderson P, Bondesson U, Mattiasson I, Johansson BW. Verapamil and norverapamil in plasma and breast milk during breast feeding. Eur J Clin Pharmacol. 1987;31(5):625-7. https//doi.org/10.1007/bf00606644.
67. Olsson B, Bolme P, Dahlstrom B, Marcus C. Excretion of noscapine in human breast milk. Eur J Clin

Pharmacol. 1986;30(2):213-5. https//doi.org/10.1007/BF00614306.

68. Gardiner SJ, Kristensen JH, Begg EJ, Hackett LP, Wilson DA, Ilett KF, et al. Transfer of olanzapine into breast milk, calculation of infant drug dose, and effect on breast-fed infants. Am J Psychiatry. 2003;160(8):1428-31. https//doi.org/10.1176/appi.ajp.160.8.1428.
69. Hill RC, McIvor RJ, Wojnar-Horton RE, Hackett LP, Ilett KF. Risperidone distribution and excretion into human milk: case report and estimated infant exposure during breast-feeding. J Clin Psychopharmacol. 2000;20(2):285-6. https//doi.org/10.1097/00004714-200004000-00036.
70. Plante L, Ferron GM, Unruh M, Mayer PR. Excretion of pantoprazole in human breast. J Reprod Med. 2004;49(10):825-7.
71. Matheson I, Samseth M, Løberg R, Faegri A, Prentice A. Milk transfer of phenoxymethylpenicillin during puerperal mastitis. Br J Clin Pharmacol. 1988;25(1):33-40. https//doi.org/10.1111/j.1365-2125.1988.tb03279.x.
72. Pütter J, Held F. Quantitative studies on the occurrence of praziquantel in milk and plasma of lactating women. Eur J Drug Metab Pharmacokinet. 1979;4(4):193-8. https//doi.org/10.1007/bf03189426.
73. Ryu RJ, Easterling TR, Caritis SN, Venkataramanan R, Umans JG, Ahmed MS, et al. Prednisone Pharmacokinetics During Pregnancy and Lactation. J Clin Pharmacol. 2018;58(9):1223-32. https//doi.org/ 10.1002/jcph.1122.
74. Lockwood PA, Pauer L, Scavone JM, Allard M, Mendes da Costa L, Alebic-Kolbah T, et al. The Pharmacokinetics of Pregabalin in Breast Milk, Plasma, and Urine of Healthy Postpartum Women. J Hum Lact. 2016;32(3):Np1-np8. https//doi.org/10.1177/0890334415626148.
75. Bauer JH, Pape B, Zajicek J, Groshong T. Propranolol in human plasma and breast milk. Am J Cardiol. 1979;43(4):860-2. https//doi.org/10.1016/0002-9149(79)90090-0.
76. Kampmann JP, Johansen K, Hansen JM, Helweg J. Propylthiouracil in human milk. Revision of a dogma. Lancet. 1980;1(8171):736-7. https//doi.org/10.1016/s0140-6736(80)91233-7.
77. Findlay JW, Butz RF, Sailstad JM, Warren JT, Welch RM. Pseudoephedrine and triprolidine in plasma and breast milk of nursing mothers. Br J Clin Pharmacol. 1984;18(6):901-6. https//doi.org/10.1111/j.1365-2125.1984.tb02562.x.
78. Rampono J, Kristensen JH, Ilett KF, Hackett LP, Kohan R. Quetiapine and breast feeding. Ann Pharmacother. 2007;41(4):711-4. https//doi.org/10.1345/aph.1H555.
79. Begg EJ, Robson RA, Gardiner SJ, Hudson LJ, Reece PA, Olson SC, et al. Quinapril and its metabolite quinaprilat in human milk. Br J Clin Pharmacol. 2001;51(5):478-81. https//doi.org/10.1046/j.1365-2125.2001.01327.x.
80. Kearns GL, McConnell RF, Jr., Trang JM, Kluza RB. Appearance of ranitidine in breast milk following multiple dosing. Clin Pharm. 1985;4(3):322-4.
81. Hackett LP, Ilett KF, Rampono J, Kristensen JH, Kohan R. Transfer of reboxetine into breastmilk, its plasma concentrations and lack of adverse effects in the breastfed infant. Eur J Clin Pharmacol. 2006;62(8):633-8. https//doi.org/10.1007/s00228-006-0140-0.
82. Gardiner SJ, Begg EJ, Zhang M, Hughes RC. Transfer of rofecoxib into human milk. Eur J Clin Pharmacol. 2005;61(5-6):405-8. https//doi.org/10.1007/s00228-005-0937-2.
83. Stoehr GP, Juhl RP, Veals J, Symchowicz S, Gural R, Lin C, et al. The excretion of rosaramicin in

breast milk. J Clin Pharmacol. 1985;25(2):89-94. https//doi.org/10.1002/j.1552-4604.1985.tb02807.x.

84. Lassman HB, Puri SK, Ho I, Sabo R, Mezzino MJ. Pharmacokinetics of roxithromycin (RU 965). J Clin Pharmacol. 1988;28(2):141-52. https//doi.org/10.1002/j.1552-4604.1988.tb05738.x.

85. Egan PC MC, Heyl PS et al. . Saccharin excretion in mature human milk. Drug Intell Clin Pharm. 1984;18:511.

86. Wojnar-Horton RE, Hackett LP, Yapp P, Dusci LJ, Paech M, Ilett KF. Distribution and excretion of sumatriptan in human milk. Br J Clin Pharmacol. 1996;41(3):217-21. https//doi.org/10.1111/j.1365-2125.1996.tb00185.x.

87. Chaikin P, Chasin M, Kennedy B, Silverman BK. Suprofen concentrations in human breast milk. J Clin Pharmacol. 1983;23(8-9):385-90. https//doi.org/10.1002/j.1552-4604.1983.tb02752.x.

88. Zheng S, Easterling TR, Hays K, Umans JG, Miodovnik M, Clark S, et al. Tacrolimus placental transfer at delivery and neonatal exposure through breast milk. Br J Clin Pharmacol. 2013;76(6):988-96. https//doi.org/ 10.1111/bcp.12122.

89. Lönnerholm G, Lindström B. Terbutaline excretion into breast milk. Br J Clin Pharmacol. 1982;13(5):729-30. https//doi.org/10.1111/j.1365-2125.1982.tb01444.x.

90. Andersen LW, Qvist T, Hertz J, Mogensen F. Concentrations of thiopentone in mature breast milk and colostrum following an induction dose. Acta Anaesthesiol Scand. 1987;31(1):30-2. https//doi.org/ 10.1111/j.1399-6576.1987.tb02515.x.

91. Sagraves R, Waller ES, Goehrs HR. Tolmetin in breast milk. Drug Intell Clin Pharm. 1985;19(1):55-6. https//doi.org/10.1177/106002808501900115.

92. Verbeeck RK, Ross SG, McKenna EA. Excretion of trazodone in breast milk. Br J Clin Pharmacol. 1986;22(3):367-70. https//doi.org/10.1111/j.1365-2125.1986.tb02903.x.

93. Darwish M, Martin PT, Cevallos WH, Tse S, Wheeler S, Troy SM. Rapid disappearance of zaleplon from breast milk after oral administration to lactating women. J Clin Pharmacol. 1999;39(7):670-4. https//doi.org/10.1177/00912709922008308.
